# Protocol for contactless and instantaneous line-current data exchange between MATLAB and a drone-deployable sensor on overhead transmission lines

**DOI:** 10.1016/j.xpro.2025.104114

**Published:** 2025-09-27

**Authors:** Khaled Osmani, Munira Halimjanova, Detlef Schulz

**Affiliations:** 1 Department of Electrical Engineering, Helmut Schmidt University, 22043 Hamburg, Germany

**Keywords:** Physics, Energy

## Abstract

Here, we present a protocol for fabricating a drone-deployable electric current sensor for real-time, remote monitoring of electric currents in overhead transmission lines. We describe steps for designing and manufacturing the board in Autodesk EAGLE and the signal processing units. We then detail procedures for programming routines on the Arduino Due to process data and curve visualization environment for Bluetooth-received data in MATLAB. The system supports data exchange up to 60 meters, contributing to the advancement of electric grid digitalization.

For complete details on the use and execution of this protocol, please refer to Osmani et al.^1^

## Highlights

• Procedures for realization of printed circuit boards and functional description thereof
• Instructions for programming Arduino Due as data processor and transmitter module
• Framework for MATLAB scripting for reception and demodulation of signal data therefrom

## Abstract (editor highlights)

Publisher’s note: Undertaking any experimental protocol requires adherence to local institutional guidelines for laboratory safety and ethics.

## Graphical abstract

**Figure:**
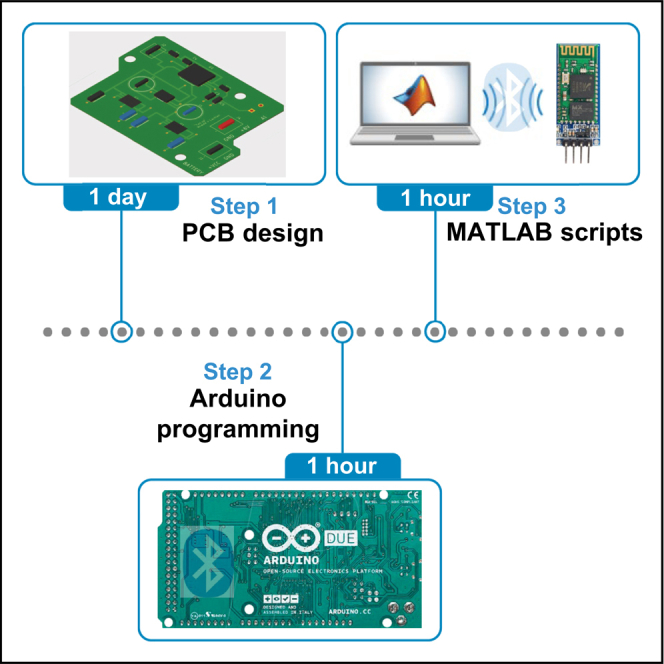

## Before you begin

This protocol outlines a step-by-step approach for developing a drone-deployable electric sensor, enabling remote monitoring of overhead Transmission Lines (TLs). The objective of this protocol is to facilitate the visualization of the Root Mean Square (RMS) values of electric currents flowing through TLs on a remote computer. With instantaneous timestamps, the RMS currents can be visualized in MATLAB installed on a remote computer positioned beneath the TL. Accordingly, utilizing the technical aspects described in this protocol, a user with MATLAB software can graphically observe the RMS currents in TLs in real time, for distances up to 60 meters (line-of-sight between the computer and the TL). The proposed sensor is housed within a dedicated sensor box designed to implement the suggested remote sensing technology. The raw data of electric currents are captured using linear Hall-effect sensors (i.e., SS494B) mounted inside fastening tubes, which are specifically designed to secure the sensor box onto the TL. The resulting Magnetic Fields (MFs) are then processed through Low-Pass Filters (LPFs) and Voltage Dividers (VDs), allowing for safe injection into the Analog Inputs (AIs) of the Arduino Due. After performing the necessary mathematical computations, the voltage values received from the Hall-effect sensors are iteratively converted back to their corresponding MFs, and subsequently to the inducing current values. Once the current values are available in the Arduino Due, they are transmitted via an HC-06 Bluetooth module to the remote computer, which is equipped with a Bluetooth signal enhancer. The received data are saved in the MATLAB workspace and graphically displayed as curves representing current norms [RMS] versus the time of data reception [hh:mm:ss].

In general, measuring electric currents in overhead TLs presents certain challenges, as such measurements are traditionally performed by service providers through trained personnel. Although more advanced technologies, such as Supervisory Control and Data Acquisition (SCADA) systems,^2^^,^^3^ Internet of Things (IoT)-based systems,^4^ and other MF-based techniques,^5^^,^^6^^,^^7^ have emerged to enhance the reliability of current measurements in TLs, none provide the same level of dynamic and remote measurement accuracy as the technique described herein. In contrast, this protocol utilizes the growing application of drones, employing the proposed sensor box as a drone payload to be deployed onto or removed from TLs. This facilitates contactless current measurement and enables real-time data transmission to a remote computer within a 60-meter range. Therefore, the protocol introduces a user-friendly approach for the on-demand visualization of electric currents in overhead TLs, offering a plug-and-play mechanism that supports grid monitoring and contributes to the advancement of smart grid technologies.

**Figure 1:**
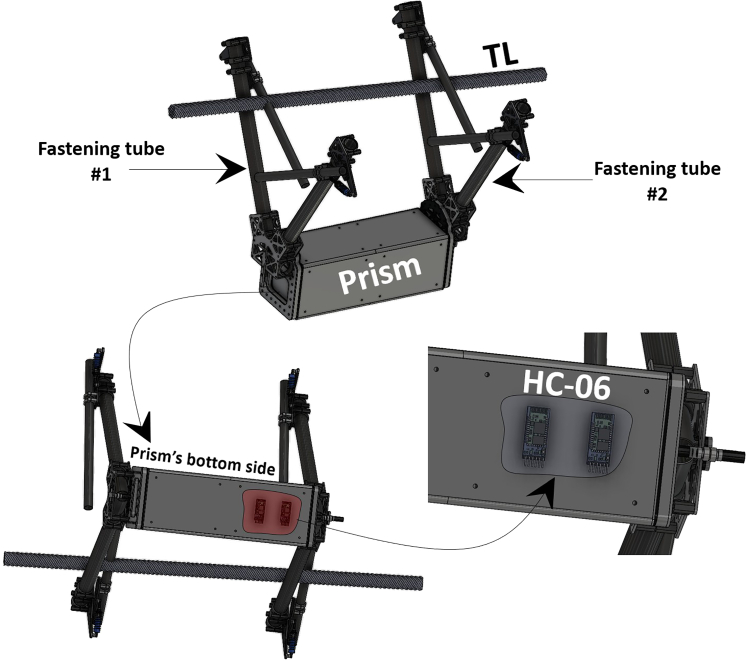
Computer-aided design of the sensor box with bottom view of the prism over which the Bluetooth HC-06 modules are engraved and fixed

***Note:*** Since the sensor box, once deployed by the drone, may fixate in two possible orientations on the TL, identical Hall-effect sensors are installed in the adjacent tubes, labeled as fastening tube #1 and fastening tube #2 in the Computer Aided Design (CAD) of the sensor box in Figure 1, and on the physical sensor box in Figure 3.

**CRITICAL:** The protocol is applicable to the sensor box presented in Figure 1, which possesses the specified material, geometric, and dimensional characteristics.

***Note:*** The applicability of the protocol presented herein depends on the geometric structure of the sensor box shown in Figure 1. More importantly, the raw material of the sensor housing (i.e., Carbon Fiber (CF)) is essential for fabricating similar enclosures, as it allows penetration of the MFs induced by currents in TLs without alteration. However, CF obstructs Bluetooth signal transmission, which necessitated the incorporation of two dedicated slots for the HC-06 modules, as illustrated in Figure 1. These modules are positioned at the bottom side of the prism to maximize their distance from the transmission lines’ vicinity, as proximity may also interfere with their emission range.

***Note:*** The CF box shown in Figure 1 must have prism dimensions of 90 mm × 300 mm × 90 mm to fully accommodate all Signal Processing Units (SPUs) presented in this protocol.

***Note:*** Each of the fastening tubes shown in Figure 1 (i.e., #1 and #2) contains two Hall-effect sensors (SS494B) inserted to capture the emitted MFs.

***Note:*** Two articulated joints, positioned in parallel on the right and left sides of the prism, serve to connect the fastening tubes shown in Figure 1 to the prism using 4 mm screws and nuts.

**Figure 2:**
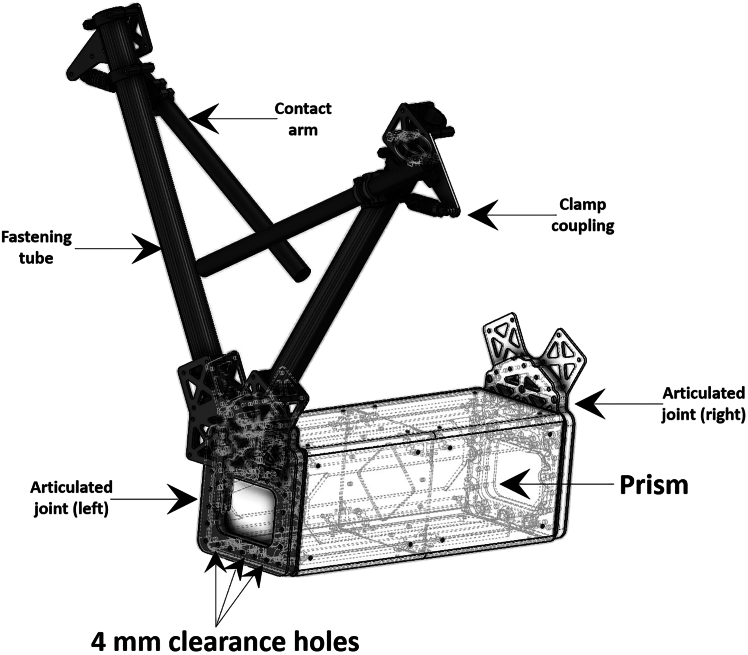
Discretized components of the sensor box and their mechanical connections

***Note:*** On the bottom side of the prism, the slots for the HC-06 modules measure 18 mm × 43 mm, with a spacing of 16 mm between them, as shown in Figure 4.

***Note:*** A contact arm connects to each fastening tube through clamp coupling as shown in Figure 2, and represents the direct contact with the TL.

**Figure 3:**
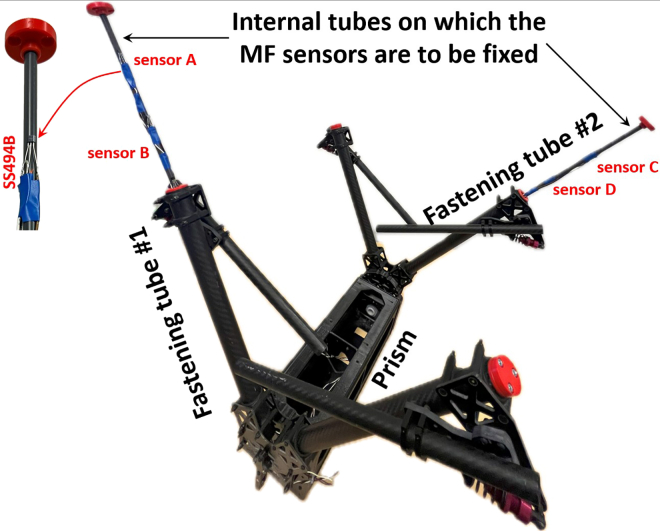
MF sensors fixation inside each of the fastening tubes

***Note:*** The output wires of each MF sensor, secured within each fastening tube, are routed into the prism housing the Arduino Due, along with the other SPUs and the power supply battery. The internal barrels shown in Figure 3, onto which the MF sensors are mounted, are mechanically dynamic, allowing flexible insertion and removal, and are easily detachable.

***Note:*** The HC-06 module on the right side in Figure 4 serves as a backup in the event of failure of the primary HC-06 module (located on the left side and activated during the experiment). Additionally, the protocol incorporates the installation of two HC-06 modules to enable the potential future transfer of further TL-related data, such as the TL’s temperature. Both HC-06 modules are oriented downward, maintaining a direct line of sight with the remote computer running MATLAB.

**Figure 4:**
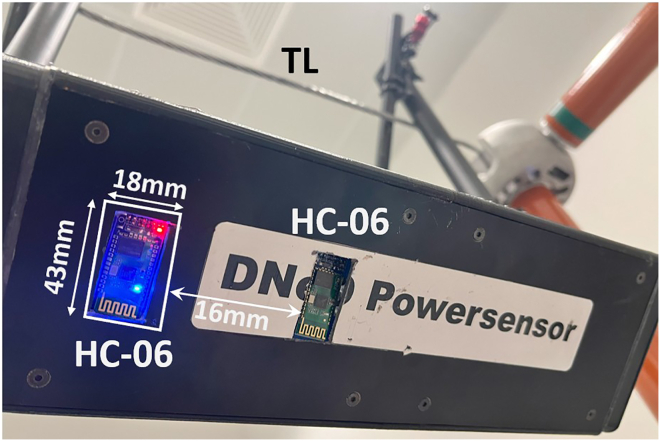
Engraving and mounting of the HC-06 modules on the underside of the sensor box

### Innovation

This protocol introduces a novel drone-deployable sensor box for remote current measurement on overhead TLs. Unlike SCADA- or IoT-based systems^2^^,^^3^^,^^4^ and existing magnetic or optical sensing techniques,^5^^,^^6^^,^^7^^,^^8^ our approach employs a drone solely as a messenger, delivering an encapsulated sensor unit directly onto the TL without requiring permanent infrastructure or complex installation. The encapsulant functions as a self-contained, transportable measurement tool that establishes Bluetooth communication with a ground computer, enabling real-time acquisition and instantaneous MATLAB visualization of current versus time for ranges up to 60 m. This integration of drone-assisted deployment with wireless data streaming represents the first demonstration of a fully portable, plug-and-play system for rapid current monitoring in overhead TLs.

## Key resources table

**Table:** 

REAGENT or RESOURCE	SOURCE	IDENTIFIER
**Software and algorithms**		

MATLAB R2023a	MathWorks	https://de.mathworks.com/products/matlab.html
Autodesk EAGLE	Autodesk	https://www.autodesk.com/products/eagle/overview
SolidWorks 2022	Dassault Systèmes SolidWorks Corporation	https://www.solidworks.com/
Arduino IDE	Arduino.cc	https://www.arduino.cc/en/software

**Other**		

Magnetic field sensor	Honeywell	SS494B
Thin film resistors	Panasonic	ERA-8AEB4322V
Wire-to-board terminal block	Molex	39773-003
Wire-to-board terminal block	PHOENIX CONTACT	1729128
Multilayer ceramic capacitors	KEMET	C1206C104F5JAC7800
Active filters 5th order	Analog Devices	LTC1062CSW#TRPBF
LDO voltage regulator (5 V)	ROHM Semiconductor	BD50GA5MEFJ-LBH2
LDO voltage regulator (8 V)	ROHM Semiconductor	BD80GC0VEFJ-ME2
Tantalum capacitor	KYOCERA AVX	TAJW225K050RNJ
Switching voltage regulators CMOS monolithic voltage converter	Analog Devices/Maxim Integrated	MAX660ESA+T
Aluminum electrolytic capacitors	Panasonic	ECE-A1VN101U
Multilayer ceramic capacitors	KEMET	C1206C751F5GACTU
Thin film resistors	YAGEO	RT1206BRD0750KL
Bluetooth modules - 802.15.1 HC-06	OSEPP Electronics	BTM-01G
Bluetooth 5.3 USB adapter with antenna	LOGILINK	BT0067
Custom-designed PCB	This paper	N/A
3S1P (12 V) 5,000 mAh battery	ENERPROF	https://enerprof.de/products/enerpower-3s1p-11-1v-akku-12v-5000mah-55wh-open-end-kabel-3x1
Arduino Due	Arduino.cc	A000062
Pluggable terminal block	WAGO	221-2411
Pushbutton switch illuminated	EAO	82-6851.1134
Internal barrels	Helmut-Schmidt-University central workshop	N/A
Cable shielding	Helmut-Schmidt-University central workshop	N/A
Entire CF sensor box housing (including the prism, fastening tubes, and connectors)	Emqopter GmbH	N/A
Screws of different widths (4, 5, and 6 mm)	Helmut-Schmidt-University central workshop	N/A
Shielded twisted pair cable	M5Stack Technology Co., Ltd.	A088-B

## Step-by-step method details

### Board design and manufacturing in Autodesk EAGLE

**Timing: 1 day**

In this first major step, we aim for the full fabrication of the Printed Circuit Boards (PCBs) used in the physical sensor. These PCBs are used as signal processing units between the MF sensors (SS494B) and the Arduino Due.

1. Using Autodesk EAGLE.
  a. Visit the Autodesk EAGLE free-download website: http://www.autodesk.com/products/eagle/free-download.
    ***Note:*** Despite the restrictions of the EAGLE free version, it is sufficient for the realization of the PCBs in this protocol.
  b. Select the appropriate version for your system (e.g., Windows, macOS).
  c. Run the installer and follow the on-screen instructions.
  d. Accept the license agreement and choose the default installation settings.
  e. Create or log into your Autodesk account.
  f. Sign in and choose the personal use type of license.
  g. Open Autodesk EAGLE and go to the control panel window.
  h. Click on File > New > Project.
  i. In the project tree, right click on the created project > New > Schematic.
2. Download the libraries needed for PCB circuit arrangement.
  a. Download the library needed for the component LTC1062CSW#TRPBF from https://app.ultralibrarian.com/details/df5cbc8c-cfed-11e9-b85e-0ad2c9526b44/Analog-Devices-Inc/LTC1062CSW-TRPBF?uid=88964761.
  b. Download the library needed for the component MAX660ESA+T from https://app.ultralibrarian.com/details/9a528f28-10a1-11e9-ab3a-0a3560a4cccc/Analog-Devices-Maxim-Integrated/MAX660ESA-T?uid=124223714.
  c. Download the library needed for the component BD50GA5MEFJ-LBH2 from https://app.ultralibrarian.com/details/821c2635-fd49-11e9-a124-0ad2c9526b44/ROHM-Semiconductor/BD50GA5MEFJ-LBH2?uid=61925775.
  d. Download the library needed for the component BD80GC0VEFJ-ME2 from https://componentsearchengine.com/part-view/BD80GC0VEFJ-ME2/ROHM%20Semiconductor.
  e. Download the library needed for the component TAJW225K050RNJ from https://componentsearchengine.com/part-view/TAJW225K050RNJ/Kyocera%20AVX.
  f. Download the library needed for the component ECE-A1VN101U from https://componentsearchengine.com/part-view/ECE-A1VN101U/Panasonic.
  g. Download the library needed for the component 1729128 from https://app.ultralibrarian.com/details/5a4d2b03-070b-11ea-a124-0ad2c9526b44/Phoenix-Contact/1729128?uid=4366.
  h. Download the library needed for the component RT1206BRD0750KL from https://componentsearchengine.com/part-view/RT1206BRD0750KL/YAGEO.
  i. Download the library needed for the component C1206C751F5GACTU from https://app.ultralibrarian.com/details/c53358a5-1072-11e9-ab3a-0a3560a4cccc/Kemet/C1206C751F5GACTU?uid=17138297.
  j. Download the library needed for the component C1206C104F5JAC7800 from https://componentsearchengine.com/part-view/C1206C104F5JAC7800/KEMET.
  k. Download the library needed for the component ERA-8AEB4322V from https://componentsearchengine.com/part-view/ERA-8AEB4322V/Panasonic.
  l. Download the library needed for the component 39773-0003 from https://app.ultralibrarian.com/details/5173117d-c817-11ea-b5d0-0aebb021a1ea/Molex-Connector-Corporation/397730003?uid=95432563.

***Note:*** The challenge in this task lies in the possibility that certain libraries may no longer be available for download and import into Autodesk. If that is the case, users must consult the technical datasheet of the missing component provided in the Key resources table and manually create an equivalent part based on the geometrical dimensions specified in the datasheet. At the time of experimentation, all necessary components were available from the specified web links.

3. Circuit arrangement in Autodesk EAGLE.
  ***Note:*** The probabilistic failure associated with this step has the potential solution for Problem 1 in the Troubleshooting section.
  a. Import all downloaded libraries into the project.
  b. Insert each corresponding component in the schematic.
  c. Generate/switch to board.
  d. Arrange all components in the specific drawing sheet.
    **CRITICAL:** Make sure that the board is designed in a two-layer configuration (i.e., top and bottom).
  e. Place the wire connectors, such as 39773-0003 and 1729128, at the outer edges of the board to facilitate future cabling.
  f. Connect the output wires of each SS494B (running from the fastening tubes to the sensor box prism) to its corresponding 39773-0003 connector on each board.
    **CRITICAL:** For the bottom layer (i.e., GND), the trace width must be at least 50 mil. For the top layer (i.e., Signal), the trace width should be at least 30 mil. Vias should have a minimum drill size of 14 mil.
  g. Connect one terminal of the 39773-0003 to +5V, second terminal to GND, and third terminal to ERA-8AEB4322V.
  h. Connect the second terminal of ERA-8AEB4322V to C1206C104F5JAC7800. From the same node, connect this branch to the OUT pin of LTC1062CSW#TRPBF and to one terminal of RT1206BRD0750KL.
  i. Connect the other terminal of C1206C104F5JAC7800 to the FB pin of LTC1062CSW#TRPBF.
  j. Connect the AGND pin of the LTC1062CSW#TRPBF to GND and the COSC pin of the LTC1062CSW#TRPBF to GND through C1206C751F5GACTU.
  k. Connect the V- pin of the LTC1062CSW’TRPBF to -5V.
  l. Connect the DIVIDERRATIO and V+ pins of the LTC1062CSW#TRPBF to +5V.
  m. Connect the remaining pin of the RT1206BRD0750KL (engaged in part (h) of this sub-task) to the other RT1206BRD0750KL, and from the same node to the 1729128.
    ***Note:*** The common node of part (m) of this sub-task is to be connected to the Arduino Due AI.
  n. Connect The other remaining terminal of the RT1206BRD0750KL to GND.
  o. Connect the V+ pin of the MAX660ESA+T to +5V.
  p. Connect the GND and LV pins of the MAX660ESA+T to GND.
  q. Connect the OUT pin of the MAX660ESA+T to GND through ECE-A1VN101U.
  r. Connect the CAP+ pin to the CAP- pin of the MAX660ESA+T through ECE-A1VN101U.
    ***Note:*** The boundary placed 1729128 components are to have two pins connected to GND, and the remaining pin of the first component to +VCC, the other of the second component to Arduino Vin.
  s. Connect the VO and VCC of both BD50GA5MEFJ-LBH2 and BD80GC0VEFJ-ME2 to GND, each through TAJW225K050RNJ.
  t. Connect the GND and EP pins of both BD50GA5MEFJ-LBH2 and BD80GC0VEFJ-ME2 to GND.
  u. Connect the FB/VO_S pin to VO in both BD50GA5MEFJ-LBH2 and BD80GC0VEFJ-ME2.
  v. Connect the EN pin to VCC in both BD50GA5MEFJ-LBH2 and BD80GC0VEFJ-ME2.
    **CRITICAL:** In precedent steps, GND should be common and originated from the GND of the 3S1P (12V) 5000 mAh battery.
  w. Conduct a Design Rule Check (DRC) and make sure that the design is error-free.
4. PCB fabrication.
  ***Note:*** The probabilistic failure associated with this step has the potential solution for problem 2 and problem 3 in the troubleshooting section.
  a. Import Gerber, drill, and netlist files into CAM350.
  b. Run DFM checks (Valor NPI) for track widths, clearances, drill sizes (≥ 0.3 mm), and outline accuracy.
  c. Plot copper, solder mask, and silkscreen layers with photoplotter (± 25 μm alignment).
  d. Laminate photoresist on FR-4, expose UV, develop, etch.
  e. Inspect (AOI), then press into multilayer stack.
  f. Drill holes (CNC), apply electroless copper, and electroplate to ∼ 25 μm.
  g. Image, electroplate, and etch outer traces (35–70 μm).
  h. Apply solder mask, expose openings, print silkscreen (min. 0.15 mm width).
  i. Add ENIG, test continuity (flying probe), route edges, and inspect.
5. Soldering.
  ***Note:*** The probabilistic failure associated with this step has the potential solution for problem 4 in the troubleshooting section.
  a. Solder paste application.
    i. Secure PCB in stencil printer, align stencil over pads.
    ii. Spread solder paste evenly with squeegee, then lift stencil for precise deposits.
  b. Component placement.
    i. Load PCB into pick-and-place machine.
    ii. Verify alignment via fiducials and optical recognition.
  c. Transfer PCB through reflow oven to melt and bond solder paste.
  d. Quality inspection.
    i. Scan for defects using AOI (e.g., misalignments, solder bridges).
    ii. Inspect BGA joints via X-ray.
    iii. Test electrical functionality.
6. Full electronic assembly.
  ***Note:*** The probabilistic failure associated with this step has the potential solution for problem 5 in the troubleshooting section.
  a. Connect the GND of the Arduino Due with the common GND of the circuit.
  b. Feed the two HC-06 modules from the +5V port on the Arduino Due header.
  c. Connect the GND of the two HC-06 modules to the common GND of the circuit.
  d. Connect the RX of the first HC-06 to TX1 of the Arduino Due. Connect the TX of this same module to RX1 of the Arduino Due.
  e. Connect the RX of the second HC-06 to TX3 of the Arduino Due. Connect the TX of this same module to RX3 of the Arduino Due.
    ***Note:*** The output pin of 1729128 (connecting the two RT1206BRD0750KL) of each PCB is dedicated for a unique AI on the Arduino Due.
  f. Connect sensor A output (after the signal processing) to AI0 of the Arduino Due.
  g. Connect sensor B output (after the signal processing) to AI1 of the Arduino Due.
  h. Connect sensor C output (after the signal processing) to AI2 of the Arduino Due.
  i. Connect sensor D output (after the signal processing) to AI3 of the Arduino Due.
    ***Note:*** Sensor A, B, C, and D refer each to the SS494B which is fixed inside the two adjacent fastening tubes of the sensor box shown in Figure 2.
  j. Detach the GND of the 3S1P (12V) 5000 mAh from the common GND of all circuitries through a single pole single throw switch.
  k. Connect the +VCC of the 3S1P (12V) 5000 mAh to the +VCC pin of each PCB.
  l. Feed the Arduino Due Vin pin from the +8V output of BD80GC0VEFJ-ME2.
  m. Connect the 3-wire terminals originated from each SS494B (in each of the two fastening tubes of Figure 2) to the corresponding pin of 39773-0003 on each PCB (i.e., Vcc connects to +5V, GND to GND, and Vout to the third terminal which is connected to ERA-8AEB4322V).
  n. Before current measurement, switch the single pole single throw to turn on the entire circuit.

### Arduino programming

**Timing: 60 min**

In this second major step, we program the Arduino Due to begin reading the analog values received on each AI after they have been processed by the SPU described in the first major step. When the Arduino Due receives a specific command via Bluetooth from the MATLAB script, it starts reading and transmitting the physical values from each AI to MATLAB.

>const int samplecount = 1000;

>const float vref = 3.3;

>const float resolution = 4096;

>int analogvalue = 0;

>float voltage = 0;

>float maxvoltage = 0

7. Start with variables definition.
  ***Note:*** No libraries are required for this specific sketch, since we are using built-in functionalities.
  a. Define sensor pins, sampling count, reference voltage and ADC resolution.
  b. Define variables for analog values, voltage, and max voltage.
    ***Note:*** The ‘samplecount’ is set to 1000 to ensure sufficient coverage of the analog signal and reliably capture its peak value. A high number of samples increases the probability of detecting transient peaks and reduces the influence of random noise. This choice hence provides a practical balance between measurement accuracy, processing time, and system responsiveness.
8. System initialization.
  a. Start serial communication for monitor (Serial) and two HC-06 modules (Serial3 and Serial1).
  b. Set the baud rate to 9600.
  c. Set analog resolution to 12 bits.

>void setup() {

>Serial.begin(9600);

>Serial3.begin(9600);

>Serial1.begin(9600);

>analogReadResolution(12);

>}

***Note:*** The baud rate is set at 9600 to ensure stable and reliable Bluetooth communication between the Arduino Due and the computer’s Bluetooth adapter: 9600 baud is a widely supported standard speed that minimizes transmission errors, especially over wireless links where interference or signal loss can occur. It also ensures compatibility with most Bluetooth modules, which typically default to this rate. The analog read resolution is configured to 12 bits to fully utilize the native 12-bit ADC capability of the Arduino Due. This setting allows analog readings to have values from 0 to 4095, providing higher precision and better signal representation compared to the default 10-bit resolution.

>float readanalog(int pin) {

>float maxvoltage = 0.0;

>for (int i = 0; i < samplecount; i++) {

>analogvalue = analogRead(pin);

>voltage = (analogvalue ∗ vref) / resolution;

>if (voltage > maxvoltage) {

>maxvoltage = voltage;

>}

>delayMicroseconds(100);

>}

>return maxvoltage;

>}

9. Read sensor data.
  a. Read analog values from AIs.
  b. Convert ADC values to voltages.
  c. Track maximum voltage over samplecount readings.

***Note:*** In the presence of an alternating MF (produced by the flow of currents in TLs), the SS494B linear Hall-effect sensor produces a corresponding sinusoidal voltage output centered around Vcc/2. This output reflects the instantaneous magnetic flux density, with amplitude proportional to the field strength and the sensor’s sensitivity. Therefore, the peak voltage of the SS494B’s AC output waveform reflects the peak magnetic field, and thus the actual current amplitude. Accordingly is the track of the maximum voltage in the Arduino IDE code related to this step.

10. Data saving and communication.
  a. Listen for the keyword “READ” from Serial3 and “READ2” from Serial1.
    ***Note:*** In this protocol, two HC-06 modules are connected to serial ports 1 and 3 of the Arduino Due (TX1/RX1 (pins 18 and 19 respectively) and TX3/RX3 (pins 15 and 15 respectively)). Only one serial port is needed for the actual measurement, with the other serving as a backup in case of a failure of the primary module. For future work, the secondary module can also transmit additional data, such as the temperature of the overhead TL.
  b. Read all 4 analog channels.
  c. Send values as space-separated string to the corresponding Serial port.
  d. Output the same to Serial monitor for debugging.
    >void loop() {
    >if (Serial3.available()) {
    >String command = Serial3.readStringUntil('\n');
    >if (command == "READ") {
    >float A0_value = readanalog(A0);
    >float A1_value = readanalog(A1);
    >float A2_value = readanalog(A2);
    >float A3_value = readanalog(A3);
    > String datatosend = String(A0_value) + " " + String(A1_value) + " " + String(A2_value) + " " + String(A3_value) + "\n";
    >Serial3.print(datatosend);
    >Serial.print("Sent Data: ");
    >Serial.print(datatosend);
    >}
    >}
    >if (Serial1.available()) {
    >String command = Serial1.readStringUntil('\n');
    >if (command == "READ2") {
    >float A0_value = readanalog(A0);
    >float A1_value = readanalog(A1);
    >float A2_value = readanalog(A2);
    >float A3_value = readanalog(A3);
    >String datatosend = String(A0_value) + " " + String(A1_value) + " " + String(A2_value) + " " + String(A3_value) + "\n";
    >Serial1.print(datatosend);
    >Serial.print("Sent Data: ");
    >Serial.print(datatosend);
    >}
    >}
    >}

### MATLAB programming

**Timing: 60 min**

The analog reading process in the Arduino Due is initiated upon receiving the syntax “READ” (or “READ2,” used here as an example to illustrate the role of the second HC-06 module). This command is sent from MATLAB via serial communication using the BT0067 Bluetooth antenna connected to the remote computer running MATLAB, with the same baud rate of 9600 as defined in the Arduino Due. Accordingly, the MATLAB programming routines in this protocol serve two purposes: first, to trigger the acquisition of RMS current values; and second, to receive these values, store them in the workspace, and represent them as a curve over time.

11. Initialization and pre-configuration.
  a. Reset global variables and user feedback.
  b. Clear any existing serial port objects.

>clear counter;

>disp('MEASUREMENTS IN PROGRESS...');

>elapsedtime = 0;

>if ∼isempty(instrfind)

>fclose(instrfind);

>delete(instrfind);

>end

12. Serial port configuration with HC-06 Bluetooth module.
  a. Define serial parameters.
  b. Open serial port connection.

>serialport = 'COM12';

>baudrate = 9600;

>s = serial(serialport, 'BaudRate', baudrate, 'Terminator', 'LF', 'Timeout', 50);

>fopen(s);

**CRITICAL:** The designation ‘COM12’ refers to the physical communication port allocated by the Bluetooth connection of the HC-06 module. This port must be defined in accordance with the actual port under which Bluetooth communication occurs. On a Windows-based computer, this can be identified through the Bluetooth settings. Furthermore, as this protocol involves two HC-06 modules, each must be distinctly identified to ensure the correct transmission of the corresponding syntax (i.e., “READ” or “READ2”).

13. Timer setup for periodic data acquisition.
  a. Define measurement duration.
  b. Create and configure timer object.
  c. Start timer to begin acquisition.

>totalseconds = 120;

>t = timer('ExecutionMode', 'fixedRate', 'Period', 1,

 'TasksToExecute', totalseconds);

>t.TimerFcn = {@readandsaveData, s, totalseconds};

>t.StopFcn = @(∼,∼) cleanupserial(s);

>start(t);

***Note:*** The timer in this protocol was set to 120 seconds solely as an example, intended to demonstrate the overall behavior and prototype response under real testing conditions. This timer can be configured for up to 6 hours, depending on the power dissipation of the Arduino Due and all associated electronic circuits, in relation to the 3S1P (12V) 5000 mAh battery.

14. Data read, parse, and Workspace assignment.
  a. Set a persistent counter for tracking data index.
  b. Send requests to Arduino and allow data time to arrive.
  c. Read incoming string and parse it.
  d. Store data into the base Workspace (when valid).
  e. Manage counter and update status.

>function readandsaveData(∼, ∼, s, totalseconds)

>persistent counter;

>if isempty(counter),

>counter = 1;

>end

>fprintf(s, 'READ\n');

>pause(2);

>receiveddata = fgetl(s);

>disp(['Received Data: ', receiveddata]);

>datavalues = sscanf(receiveddata, '%f %f %f %f ');

>if length(datavalues) == 4

>assignin('base', sprintf('analogvalA%d', counter), datavalues(1));

>assignin('base', sprintf('analogvalB%d', counter), datavalues(2));

>assignin('base', sprintf('analogvalC%d', counter), datavalues(3));

>assignin('base', sprintf('analogvalD%d', counter), datavalues(4));

>currenttime = datestr(now, ‘HH:MM:SS’);

>assignin('base', sprintf('actualtime%d', counter), currenttime);

>else

>disp (‘Error’);

>end

>if counter == totalseconds

>disp('...MEASUREMENTS COMPLETED');

>counter = 1;

>end

>counter = counter + 1;

>end

**CRITICAL:** In the above code, the sent syntax was “READ”. In accordance with the Arduino IDE code, this triggers only the HC-06 connected to Serial3 on the Arduino Due. Accordingly, the COM port should be precisely inputted with regards to the Bluetooth connection established with this HC-06 module.

15. Serial port cleanup after acquisition.
  a. Check validity and close serial properly.
  b. Ensures all resources are safely released.

>function cleanupserial(s)

>if isvalid(s)

>fclose(s);

>delete(s);

>clear s;

>disp('Serial connection closed.');

>else

>disp('Invalid serial object.');

>end

>end

***Note:*** The following four steps serve to visually represent, in MATLAB, the raw voltage values received from each MF sensor after being output by the corresponding SPU. These steps therefore function as a reference for additional information regarding the RMS current norms, as they also provide insight into the operational status of each MF sensor (e.g., defective, functioning normally, saturated, etc.). It is important to note that the plots generated by these steps do not represent the final RMS values.

**CRITICAL:** The four steps should be implemented in a separate script, distinct from the one above, and are intended exclusively for plotting purposes.

16. Time extraction and formatting.
  a. Get current date as string.
  b. Extract start and end measurement times from base workspace.
  c. Convert time strings to datetime objects.
  d. Decompose datetime into hour-minute-second for display.

>currentdate = datestr(now, 'yyyymmdd');

>starttime = eval('actualtime1');

>endtime = eval('actualtime120');

>startdatetime = datetime(starttime);

>enddatetime = datetime(endtime);

>[startyear, startmonth, startday, starthour, startmin, startsec] = datevec(startdatetime);

>[endyear, endmonth, endday, endhour, endmin, endsec] = datevec(enddatetime);

>starttimestr = sprintf('%02d:%02d:%02d', starthour, startmin, startsec);

>endtimestr = sprintf('%02d:%02d:%02d', endhour, endmin, endsec);

***Note:*** The start and end times were set to 1 and 120, respectively, in accordance with the two-minute timer defined in the variable ‘totalseconds’, which was only used as an example in this protocol. For other operating times (i.e., longer or shorter timer), this should be changed accordingly.

17. Data retrieval from workspace.
  a. Define range and initialize matrix.
  b. Load sensor data from workspace dynamically.
    >counterrange = 1:120;
    >analogvalues = zeros(length(counterrange), 4);
    >for i = counterrange
    >for j = 1:4
    >varname = sprintf('analogval%c%d', char('A'+j-1), i);
    >analogvalues(i, j) = evalin('base', varname);
    >end
    >end
    >actualtimes = cell(1, length(counterrange));
    >for i = counterrange
    >varname = sprintf('actualtime%d', i);
    >actualtimes{i} = evalin('base', varname);
    >end
    >actualtimes = datetime(actualtimes, 'InputFormat', 'HH:mm:ss');
  c. Load corresponding timestamps for each sample.
18. Plotting results (raw voltages from each MF sensor).
  a. Create a figure with 4 subplots.
  b. Set an overall title with date and time range.
  c. Save figure to file.

>figure;

>for j = 1:4

>subplot(2, 2, j);

>plot(actualtimes, analogvalues(:, j), '-o');

>xlabel('Time');

>ylabel(sprintf('Analog Value (Sensor %c)', char('A'+j-1)));

>title(sprintf('Sensor %c (A%d)', char('A'+j-1), j-1));

>grid on;

>end

>sgtitle(['i(t) Date: ', currentdate, ' Time: ', starttimestr, ' to ', endtimestr]);

>filename = [currentdate, '_StartTime', starttimestr, '_EndTime', endtimestr];

>filenamewithoutcolons = strrep(filename, ':', '');

>savefig(gcf, [filenamewithoutcolons, '.fig']);

**CRITICAL:** The following final steps in this third main task are to be implemented in a separate script, distinct from the two previously described scripts.

***Note:*** Based on the values received from each MF sensor (as plotted in the preceding four steps), a decision-making process is formulated through the steps below to represent the actual RMS current norm in relation to the corresponding measurement time. This decision-making process is founded on the comparison of the outputs from each MF sensor (among a total of two per fastening tube, as illustrated in Figure 2). Only the first sensor (whether sensor A or sensor C) in each fastening tube (whether #1 or #2, depending on which side the sensor box is mounted on the TL) is considered for representing the actual RMS values, up to the point of saturation. Upon saturation, its output voltage becomes constant at 4.5 V, as specified in the SS494B datasheet. With this in mind, the determination of the actual RMS current norm will then rely on the output from the succeeding MF sensor, until it likewise saturates.

***Note:*** If the sensor box is positioned on the TL at location A, the RMS calculations will be based on sensor A and sensor B (located in fastening tube #1). Conversely, if the sensor box is positioned at location B, the calculations will involve sensor C and sensor D (located in fastening tube #2). When the sensor box is in position A, sensor C and sensor D are disregarded; reciprocally, when the sensor box is in position B, sensor A and sensor B are disregarded. Overall, the sensor is capable of measuring currents up to 5 kA RMS.

19. Initializing the plotting of the actual RMS current norms.
  a. Extract time and format it into hour-minute-second for display.
  b. Retrieve data from workspace of each sensor’s data with corresponding timestamp.

***Note:*** Both sub-steps a. and b. in this step are the exact same replication of the code presented in step 16 of this protocol.

20. Define the sample counter range and initialize the output vector for analog values.

>counterrange = 1:120;

>analogvalues = zeros(length(counterrange), 4);

21. Define the threshold for zero-current detection.

>threshold = 2.5;

**CRITICAL:** The value of 2.5 is selected as a threshold based on the typical output of the Hall-effect SS494B sensor under null magnetic field conditions (i.e., no magnetic field present due to the absence of current) when supplied with +5V. This value is considered “ideal” and must be experimentally validated, particularly in the context of the CF sensor box implementation.

22. Define the mapping function (by which the raw sensor analog readings are mapped to predefined output levels).

>function mappedvalue = mapanalogvalue(analogval)

>thresholds = [*threshold1*, *threshold2*, … , *thresholdn*];

>mappedoutputs = [0, …, *maxcurrent*];

>idx = find(analogval <= thresholds, 1, 'first');

>if isempty(idx)

>mappedvalue = analogval;

>else

>mappedvalue = mappedoutputs(idx);

>end

>end

**CRITICAL:** The *thresholds* in the above code represent the actual output voltage produced by the SS494B sensor upon its exposure to the MF induced by current flow. These values can only be determined through real experimentation, as the constraints associated with the CF sensor box cannot be generalized. Therefore, the sensor described in this protocol requires a one-time calibration, during which the above code can be numerically specified. This requirement also applies to the *maxcurrent* value, as it depends on the positioning of the SS494B sensors on the internal barrels shown in Figure 3.

***Note:*** In this protocol, the core challenge lies in mapping and identifying the raw analog voltage. This difficulty arises primarily from the nearly unpredictable voltage output of each SS494B Hall-effect sensor, caused by the physical constraints imposed by the CF material and the positioning of each MF sensor on the internal barrels shown in Figure 2. Even slight modifications to the position of an SS494B sensor on the barrels lead to significant changes in the output voltage. Therefore, the positioning of each SS494B sensor must be fixed to ensure accurate readings.

23. Process all samples with sensor-priority logic.
  a. Begin the main loop to process each time step.
  b. Retrieve and map the value from sensor A.
  c. When the mapped value from sensor A equals the saturation threshold, fetch sensor B, map it, and sum the two.
  d. When the mapped value from sensor A is less than the minimal threshold, check sensor B.
  e. When both sensor A and sensor B are below the minimal threshold, check sensor C, then, after sensor C saturates, check sensor D.
  f. When both sensor C and sensor D are below the minimal threshold, set zero to the output current.
  g. When the condition in f. in this step is not met, prefer sensor C and sensor D mapping.
  h. Assign the overall current value, based on which sensor gives a valid reading.

>for i = counterrange

>varnameA = sprintf('analogvalA%d', i);

>analogvalA = evalin('base', varnameA);

>mappedA = mapanalogvalue(analogvalA);

>if mappedA == *maxcurrent*

>varnameB = sprintf('analogvalB%d', i);

>analogvalB = evalin('base', varnameB);

>mappedB = mapanalogvalue(analogvalB);

>analogvalues(i) = mappedA + mappedB;

>else

>if analogvalA < threshold

>varnameB = sprintf('analogvalB%d', i);

>analogvalB = evalin('base', varnameB);

>mappedB = mapanalogvalue(analogvalB);

>if analogvalB < threshold

>varnameC = sprintf('analogvalC%d', i);

>varnameD = sprintf('analogvalD%d', i);

>analogvalC = evalin('base', varnameC);

>analogvalD = evalin('base', varnameD);

>mappedC = mapanalogvalue(analogvalC);

>mappedD = mapanalogvalue(analogvalD);

>if analogvalC < threshold && analogvalD < threshold>analogvalues(i) = 0;

>else

>if mappedC == *maxcurrent*

>analogvalues(i) = mappedC + mappedD;

>else

>analogvalues(i) = mappedC;

>end

>end

>else

>analogvalues(i) = mappedB;

>end

>else

>analogvalues(i) = mappedA;

>end

>end

>end

24. Load all timestamps corresponding to the measurements then plot current over time.

>actualtimes = cell(1, length(counterrange));
>for i = counterrange
>varname = sprintf('actualtime%d', i);
>actualtimes{i} = evalin('base', varname);
>end
>actualtimes = datetime(actualtimes, 'InputFormat', 'HH:mm:ss');
>figure;
>plot(actualTimes, analogValues, '-o');
>xlabel('Time');
>ylabel('RMS [Amps]');
>grid on;
>sgtitle(['i(t) Date: ', currentdate, ' Time: ', starttimestr, ' to ', endtimestr]);

25. Generate a file name and save the plot for further analysis.

>filename = [currentdate, '_Starttime', starttimestr, '_EndTime', endtimestr];

>filenamewithoutcolons = strrep(filename, ':', '');

>savefig(gcf, [filenamewithoutcolons, '.fig']);

***Note:*** The saved filename includes the measurement date and the start and end times, ensuring each dataset is uniquely identified. This allows efficient organization and retrieval without opening files individually. The recorded data provides a basis for analyzing current fluctuations over time, supporting trend monitoring. With time-referenced measurements, it is possible to build a reliable database for detecting deviations, enabling predictive maintenance of electric grids and improving system reliability.

## Expected outcomes

**Figure 5:**
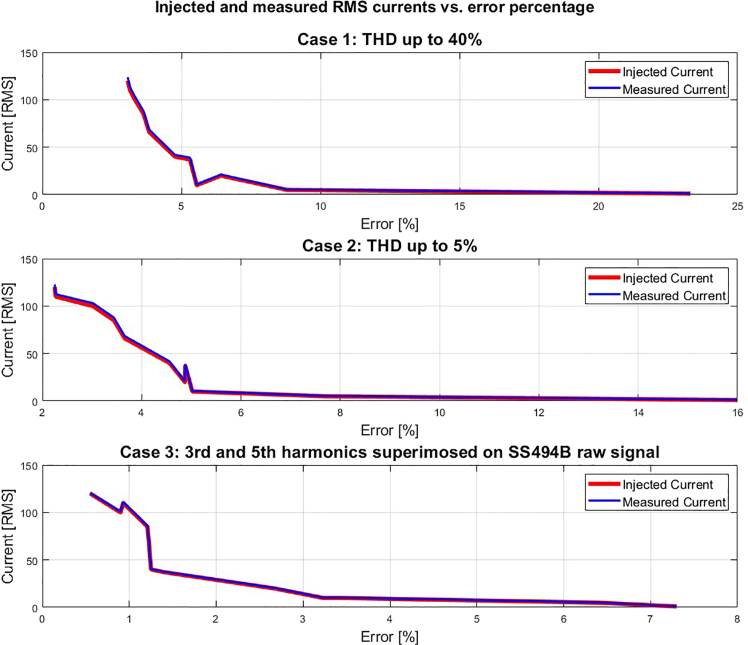
Error representation between reference and measured currents under laboratory stress conditions

Laboratory tests injecting random RMS current values would likely reveal that the sensor box performs well under dynamic fluctuations, such as increasing current from 0 ARMS to 66 ARMS within 2 seconds or decreasing from 120 ARMS to 10 ARMS in 5 seconds. Under 40% Total Harmonic Distortion (THD), errors would be expected to range from approximately 23.3% at 1 ARMS to 3.07% at 120 ARMS, with higher currents improving accuracy. With a THD of 5%, the error would likely decrease to around 16% at 1 ARMS and 2.25% at 120 ARMS, while superimposing 3rd and 5th harmonics would likely result in even lower errors, ranging from 7.3% at 1 ARMS to 0.55% at 120 ARMS. The system’s error rates would decrease as current increases, with values under 0.55% anticipated for currents exceeding 100 ARMS under typical conditions. Figure 5 presents a graphical visualization of the error between injected and measured currents under laboratory stress conditions, evaluated across three test cases: Case 1 involves injected currents with up to 40% THD; Case 2 involves currents with up to 5% THD; and Case 3 involves the superposition of third and fifth harmonics (relative to the intrinsic 50 Hz signal) onto the output signal of each SS494B MF sensor.

In terms of communication, it would be expected that the sensor maintains stable transmission within a 60-meter open-air range and up to 15 meters through physical barriers, with no gradual signal degradation observed. The sensor box, weighing less than 0.6 kg, would exert minimal load on the monitored TL, and the integrated 3S1P (12V) 5000 mAh battery would support continuous operation for approximately six hours, transmitting data at 1-second intervals to MATLAB. This functionality would enable real-time monitoring and data storage, with minimal setup required for deployment, making the system efficient for long-term operation in the field.

## Limitations

The study acknowledges several limitations in its applicability. The need for a field worker beneath the TL with MATLAB functionality may be impractical under adverse weather conditions. Extrapolation for high-current measurements beyond the sensor’s validated range of 120 ARMS introduces uncertainties, including potential non-linearities, saturation, or material degradation. This is why the sensor requires in-laboratory calibration to predict higher currents based on initial outputs, eliminating the need for re-calibration thereafter. Environmental factors such as temperature fluctuations or electromagnetic interference may also affect sensor performance at elevated currents, leading to inaccuracies in extrapolated data. While laboratory-based efficiency results were presented, these cannot be fully guaranteed for real-world TL operations, as the field test focused only on communication. The TL in the field test did not carry current, leaving uncertainties about the system’s performance under non-linear grid loads. The sensor box has limitations that affect its reliability, including challenges posed by wind during drone deployment, which may disturb sensor positioning. Sag effects in overhead TLs can alter sensor alignment, further compromising measurement accuracy. Additionally, exposure to electric fields can induce voltages in the internal wiring and PCB traces, causing noise and distortion.

Although the proposed protocol introduces an innovative approach for contactless current measurement in overhead TLs using a drone, offering high reusability and deployability without requiring a reset of the internal magnetic field (MF) sensors, it still presents several limitations when compared to existing measurement technologies. For example, the sensor readings are susceptible to interference from nearby MFs, in contrast to the optical-based sensing system^8^ which complies with IEC 61869-14 standards and demonstrates improved immunity to MF disturbances. Furthermore, the sensitivity of the proposed sensing system is highly dependent on the precise positioning of the internal sensors, unlike the robust and consistent sensitivity achieved in another sensing solution.^9^ Additionally, the physical configuration of the overhead TL (particularly its conductor thickness) poses a challenge to the applicability of the proposed sensor box across diverse geometries, a limitation not encountered in another study.^5^ Under laboratory-induced stress conditions (e.g., injected currents with a THD of up to 5%), the proposed protocol exhibited a maximum measurement error of 7.72% in Case 3 of Figure 4, which is nearly four times higher than the error reported in^10^ and twelve times greater than that in.^11^

Despite partial shielding, further improvements are required for high-voltage applications. Adjacent TLs may introduce signal interference, reducing measurement accuracy. A mitigation strategy could involve multiple sensors for differential measurements. Further testing is needed to assess data fidelity, communication range, and the sensor box’s durability, particularly after its drop from the TL. While shock absorbers were used during field tests, additional research into the sensor box’s robustness under impact is recommended.

## Troubleshooting

### Problem 1

Overheating due to small vias on wide GND traces.

### Potential solution

Use thermal reliefs and multiple larger vias near heat-sensitive components.

### Problem 2

Misalignment of solder mask openings with pads.

### Potential solution

Run precise DFM checks and ensure accurate layer registration (±25 μm) before solder mask exposure.

### Problem 3

Drill holes too small for plating or cause bit breakage.

### Potential solution

Ensure all drill sizes meet the ≥0.3 mm minimum and verify tool settings before CNC drilling.

### Problem 4

Solder bridges or insufficient joints due to uneven solder paste application.

### Potential solution

Ensure proper stencil alignment and consistent squeegee pressure during paste spreading.

### Problem 5

Interference between analog signals from multiple sensors causes erratic readings.

### Potential solution

Use decoupling capacitors of 100 nF near each sensor output and route analog lines away from digital or power traces.

## Resource availability

### Lead contact

Further requests for resources and materials should be directed to and will be fulfilled by the lead contact, Detlef Schulz (detlef.schulz@hsu.hamburg).

### Technical contact

Technical questions on executing this protocol should be directed to and will be answered by the technical contact, Khaled Osmani (alosmani.k@hsu-hh.de).

### Materials availability

This study did not generate new unique reagents.

### Data and code availability

The published article includes all code generated or analyzed during this study.

## Acknowledgments

This research paper is part of the project DNeD (“Digitalisierte, rechtssichere und emissionsarme flugmobile Inspektion und Netzdatenerfassung mit automatisierten Drohnen,” engl. “Digitalised, legally safe and low-emission airborne inspection and grid data acquisition using automated drones”) and was funded by dtec.bw—Digitalization and Technology Research Center of the Bundeswehr. dtec.bw is funded by the European Union—NextGenerationEU.

## Author contributions

K.O. was responsible for preparing the initial manuscript draft, designing the electronic boards, developing and implementing the algorithms, and conducting laboratory and field testing of the complete sensor unit. M.H. helped with the writing, review, and editing. D.S. supervised the project, oversaw its administration, and verified the results as well as the scientific validity of the work.

## Declaration of interests

The authors declare no competing interests.
